# Antiseptic Surgery

**Published:** 1877-11

**Authors:** A. C. Girard


### ANTISEPTIC SURGERY.

LISTER'S SYSTEM IS NOT THE APPLICATION OF PECULIAR DRESSINGS OR THE USE OF PARTICULAR ANTISEPTICS. It is a method of treating wounds based on the "germ theory," and its principal aim is to prevent the entrance of germs into wounds, to destroy them if already there, and to guard against the accumulation of wound-secretions. In order to attain this end he surrounds the patient with a series of precautionary measures, which have from time to time been improved on by him. These require peculiar materials and appliances, of which I present the latest phase that came under my observation. They are enumerated without particular plan. Where the mode of preparation is not indicated, it will suggest itself; where it is described it is adapted as much as possible to self-preparation, thereby facilitating the introduction of the system by diminishing its greatest objection—the cost. Their use is described as concisely as possible, and will become clearer when I shall treat of the mode of operating and dressing.

1. CARBOLIZED SOLUTION (Acid. carbol. cryst. 5, aq. 100) is used to clean the neighborhood of wounds before operation, to disinfect the hands of Surgeon and assistants, and instruments, to wash out septic wounds and clear drainage tubes.

2. CARBOLIZED WATER, a 2½ per cent solution of crystalized carbolic acid in water. It is used in the spray and to wet the "lost gauze."

3. CARBOLIZED OIL (Acid. carbol. cryst. 5, ol. oliv. 100), to oil catheters or other instruments, or fingers when about to be introduced into some of the cavities. It is also employed when a constant direct contact of the antiseptic with the wound is necessary, as in caries,—or where the gauze dressing cannot be applied, as in abscess of the rectum.

4. SOLUTION OF CHLORIDE OF ZINC (8 per cent), 1 part of liq. zinc. chlor. mixed with 3 parts of water. Where wounds have been exposed unprotected to the access of atmospheric air, or where, from mistake in dressing, aseptic wounds have become septic, they are swabbed out with this solution. It is more effective than carbolic solution, but too powerful for permanent use in the dressings.

5. THE SPRAY. In order to prevent the entrance of living germs, during an operation or dressing, a spray of "carbolized water" is directed on the wound. The best instrument for this purpose is "Lister's spray," a steam atomizer which throws a large cone of finely divided spray. It is almost indispensable in long operations and where a considerable space of tissue is exposed. In its absence it may be replaced by the ordinary steam atomizer, of which two ought to be at hand, as they are sooner exhausted. Their suction tube is unnecessary and a glass tube, drawn to a fine point, and bent at an acute angle, to throw the steam against wounds without necessity of tipping the instrument, may take its place. In the absence of these, or for short dressings, Richardson's spray apparatus may be used. It has, however, serious defects; it gives out frequently without apparent cause, is very fatiguing, and wets the wounds to much, as the spray is not as finely divided as that of the steam atomizer.

6. THE PROTECTIVE is oiled silk, coated with copal varnish, to render it impermeable, and then covered with a thin layer of 1 part dextrine, 2 parts starch, and 16 parts "carbolized solution," to facilitate adhesion of the disinfecting fluids into which it is dipped before being applied to the wound. The purpose of the "protective" is to prevent the irritating effect of the contact of the antiseptic with the wound. It is placed immediately over this, overlapping it but little.

7. THE ANTISEPTIC GAUZE, a peculiar unstarched cotton gauze, selected by Mr. Lister on account of the facility with which secretions penetrate its meshes. It is prepared with antiseptics, and thus, after absorbing the wound fluids, it prevents their decomposition. Pieces of cotton gauze, six yards in length and one yard in width, are to be placed in a zinc trough and heated in the water-bath for several hours, after which they are spread out and a hot mixture of 1 part cryst. carbolic acid, 5 common resin and 7 paraffine is poured over them by means of a syringe. They are then returned to the trough and submitted to pressure for some hours, to cause an even distribution of the fluids. The resin is to hold the carbolic acid more firmly, and prevent it from being washed out or evaporated too quickly. The paraffine diminishes adhesion of the dressing. This gauze is prepared in factories in Germany, the best known being the "International" factory, at Schaffhausen, Switzerland. Its great cost (20 cents a yard in the factory) is a considerable drawback. I have, therefore, tried to replace it by cheaper material, and to prepare it myself, and have successfully used old mosquito netting for this purpose. As this is really the only part of Lister's dressings for which the Army Supply Table does not furnish the materials, it may be of interest to learn how it is done. Old mosquito bars, which have become useless for their legitimate purpose, are steeped, or, perhaps better, boiled in lye, to remove all dirt and render them more hygroscopic. They are then immersed in the hot resin mixture, which may be heated in a tin bucket on the stove. To remove the surplus liquid they are passed through a clothes-wringer, allowed to cool, stretched into shape, and put away in a closed vessel or wrapped up in oiled muslin. The clothes-wringer is easily cleaned with boiling water and a cloth. I have used this antiseptic netting with the same results as the imported gauze.

The gauze is used wet with carbolized water in immediate contact with the "protective," folded in about six thicknesses and overlapping the "protective" several inches. The remainder of the gauze is applied dry. Lister considers eight layers, *largely overlapping* the wound, sufficient. Between the seventh and eighth layers he inserts the "McIntosh."

8. THE MCINTOSH, common rubber cloth, is used to keep the secretions of the wound from finding their way immediately to the surface, and to compel them to permeate the whole dressing, thus being constantly in contact with the carbolic acid. It is cut an inch smaller than the gauze, that the secretions when about to appear externally may be discovered, while yet lying in antiseptic material. The "McIntosh" need not be renewed with the dressing, but may be washed off with carbolized water and used again.

9. CATGUT is the *sine qua non* of Lister's dressing for ligatures and deep sutures. In a few days it is absorbed and the wounds close over it without danger of its acting as an irritant or permitting hæmorrhage. In order to prevent its becoming soft, spongy and slippery, it has to go through a process, by which it is rendered tough, flexible and able to resist the action of the wound-secretions for a sufficient length of time. It is first shaken up in a carbolized emulsion of 1 part cryst. carbolic acid (dissolved by addition of 10 per cent water), and 5 parts olive oil. It should then be no more disturbed but set aside in a cool place. The catgut should be kept separate from the watery part of the emulsion. This is most conveniently done by placing a sufficient number of pebbles into the vessel containing the emulsion to support a small disk of glass out of reach of the water, on which the coils of catgut are laid. It takes at least two months before it acquires the necessary qualities. The catgut is used in different sizes, from the thickness of a horsehair to that of twine.

10. SALICYLIC COTTON. Mr. Lister has of late used this to bolster up and fill inequalities of the surface. The salicylic acid not being volatile, and cotton easily impregnated by it, it forms a useful supplement to the gauze. It is prepared by first removing the fatty matter of the cotton, by boiling it in lye or other alkaline liquid. Thus the cotton becomes hygroscopic, which quality may easily be tested by throwing a small ball on water. In its natural state it will not sink,—if prepared it will go to the bottom of the vessel in a very short time. The proportions are about 8 ozs. of the acid, 8 ozs. alcohol, and 2 gallons of water. The addition of about 20 per cent of glycerine is said to prevent the disagreeable dusting of the acid.

11. SPONGES. All sponges used in connection with Lister's dressing, in the cleaning of wounds and absorption of wound-secretions, should be prepared in the following manner: After being beaten, cleaned and washed out in lukewarm distilled water, they are immersed in the "carbolized solution," and kept there until needed. After use they are washed out in the solution and replaced in the vessel. In this manner they can be used over again as long as they last. In addition to the usual small dressing sponges some larger ones are to be kept on hand. They are applied to wounds the first day after the operation to absorb the more copious secretion caused by the spray.

12. DRAINAGE-TUBES are small flexible rubber tubes, of the size of a small quill to that of a little finger, with numerous openings on the sides, each of half the diameter of the tube. Their use is to facilitate the egress of wound-secretions without interfering with union. One or more are introduced, according to the size and number of angles of the wound, reaching to the bone. They are secured to the skin by a thread drawn through their extremity and fastened by a strip of isinglass or a small safety-pin. They must be cut on a level with the skin, as otherwise the pressure of the dressings will obstruct their lumen. In places where the slippery rubber tube is not easily retained, as in the anus, some lint dipped in carbolized oil might take its place. Of late Mr. Lister adds to the rubber tube several threads of catgut. The tube is removed in a few days, the catgut assisting in the drainage as long as it is necessary and is then absorbed, the outside parts falling off.

13. ANTISEPTIC SILK. The catgut is only used in ligatures and deep sutures. For superficial sutures it does not retain its firmness long enough and silk is preferred in its stead. This is rendered antiseptic by being steeped for an hour in a hot mixture of beeswax 10, cryst. carbolic acid 1, and then drawn through a cloth to remove the surplus wax. It is then preserved in a closed vial. In this manner it combines the advantages of the wire with the suppleness of the silk. Septic germs cannot penetrate in the interstices of the fiber, and the wax, besides increasing the hold of the knot, retains a sufficient amount of the antiseptic to destroy any germs which might enter the suture.

I might here, also, mention a method introduced by Mr. Lister to approximate the edges of the skin where from loss of substance this would be difficult, if not impossible, by superficial sutures, as in cancer in the breast when a part of the skin is implicated and has to be removed. A piece of annealed or silver wire is passed through the whole thickness of the flap, some two or three inches from its free edge, and brought out on the opposite side at the same distance. The ends of the wire are fastened over a small lead plate, thus sufficient force can be used to cover almost any deficiency. As many of these wires may be applied as the size of the wound may necessitate. The superficial sutures are then introduced, and a union by first intention may be looked for. The secretions of the wound pass through the drainage-tube. Whenever the neighboring integument seems to have accommodated itself to the strain, the wires are removed by cutting them at one of the plates. I have used this once, with success, after removal of a tumor of the scalp, with a circular loss of skin of two inches diameter, and obtained union *per primam*.

In order to complete the list of dressings pertaining to Lister's system, I must add the preparations of boracic acid, which is claimed to be an antiseptic, milder than carbolic acid, but holding its properties longer.

1. Boracic Water ($3\frac{1}{2}$ per cent solution of boracic acid in water). The boracic acid is obtained from borax by decomposition with sulphuric acid. The water is mainly used to moisten the boracic lint, where this is employed.

2. Boracic Lint is prepared like salicylic cotton, which will be mentioned hereafter. It takes the place of carbolized dressings, where these cannot be closely adapted to the skin, as in resection of the lower jaw and in harelip sutures.

3. Boracic Ointment (Acid borac., ceræ alb., of each 10, ol. amygd. dulc., paraffine, of each 20, fiat ungt.) This is used spread on thin cotton and applied to ulcers and granulating surfaces. It is neatly fastened to the skin by painting the edges over with collodion. I have seen it used with success after ablation of epithelial cancer of the face.

In applying Lister's dressings it must be borne in mind that they promise complete success only in operations where the prescribed precautions for destruction of germs, or prevention of their entry into the wound, have been conscientiously observed. Even the most careful disinfection of wounds which have been exposed unprotected will not always be successful, as germs may have penetrated beyond the reach of the antiseptic. Still even there the probabilities are in favor of antisepsis, and the facility of evacuation of pus by the drainage-tubes, and its disinfection in the meshes of the gauze, which also prevents the entrance of new living germs, predisposes most wounds to rapid healing. We must at all times be thorougly alive to the fact that the entrance of *one* living germ may destroy the usefulness and render an operation, which otherwise would barely affect a patient's health, the starting point of erysipelas, gangrene or pyæmia. These germs may be introduced by the Surgeon's finger, by the instruments, by the very dressings, if any one of Lister's minute directions is neglected. On the other hand, if once a Surgeon and assistants understand the system thoroughly, it is the easiest mode of operating and dressing; the rules are so positive that surgery loses its speculation and a quick recovery can almost with certainty be looked for. A Surgeon in one of the large German cities told me he followed Lister's system, but did not get the vaunted results. He invited me to witness a small operation which he was going to perform "under Lister." It explained to me his want of success. While he thought he followed Lister's rules rigidly, he committed nine grave faults, each of which would nullify their benefits. Still in case of failure, he would have claimed that he failed in spite of following Lister. Such adherents are his worst enemies. The importance of the lesson may excuse this anecdote.

Whenever elevation of temperature, rapidity of pulse, erysipelas, swelling, diffuse cellulitis, symptoms of pyæmia follow an operation, we may be sure that in nine cases out of ten some fault has been committed,—either the discharge has appeared on the surface and been allowed to become septic, or the the drainage-tube is closed, or not properly trimmed, or not introduced to the proper point.

The following articles, as a rule, should be at hand before proceeding to an operation: Towels, soap and sponge with warm water; nail-brush and ether; two bottles carbolized water; two atomizers, filled—the second in case the first should fail; basin, with necessary small sponges, and one or two large ones, soaking in carbolized solution; bottle with the chloride of zinc solution, to disinfect the hands of Surgeon and assistants—the instruments and a piece of gauze should also be kept in this basin; vessel with catgut; vessel with drainage-tubes; bottle with antiseptic silk; and the necessary gauze and McIntosh.

First of all the hair in the vicinity of the place to be operated on must be carefully shaved off. The skin is then to be cleaned with soap, water and a nail-brush, and after that with ether, to remove fatty matter and detritus. The patient is then etherized. Here I must mention that in almost all cases *chloroform* is used in Germany as an anæsthetic, and in a most fearless manner, even for a painful dressing. I was informed by several distinguished Surgeons that they never had a death from chloroform in thousands of operations. It is true they had, at times, cases of partial asphyxia and collapse, but never failed, by persistent efforts, to revive the patient. One case was related to me where attempts at resurrection were continued for four hours on a patient to all appearances dead; the heart was, during that time, incited to a feeble action by means of electricity on the phrenici, and slight blows in the precordial region. He finally recovered. I may be pardoned this digression in view of the now general crusade against chloroform in this country.

As soon as the patient is thoroughly anæsthetized the spray is set in operation. Should one of the extremities be the suffering part, Esmarch's bandage and tourniquet are applied in almost all cases. The only exception I know of is where extensive suppuration is going on in the limb, when purulent matter might be forced into the circulation. In that case the limb is simply elevated and a tight tourniquet applied some distance above the seat of operation. The advantages of Esmarch's bandages are too well known to need repetition.

A final washing with carbolized solution is now given to the skin, and the spray is made to play on the wound, taking care not to allow the hand of the operator to intervene. The instruments are taken from the basin as needed and returned to it as soon as used. The blood is cleared away by the antiseptic sponges. Bloodvessels are secured with the usual forceps and ligated. The ligation in operations performed by the "bloodless method," is done before removing the tourniquet. The place of the large vessels is well known, the small ones are usually found at the intersection of the muscles. The ligation is done with the catgut, care being taken not to use too thick a cord for small vessels, and to make a reef-knot. The two ends are cut off close. The ligature is absorbed or passes off with the discharge, if there is any. Numberless experiments have shown that catgut, if prepared as mentioned above, does not relax until a safe thrombus is formed.

Should, at any time during the operation, the spray cease to work, then the piece of gauze which lays ready in the carbolized water, is to be thrown quickly over the wound, and kept there until the spray is again in proper condition.

The operation being completed, the tourniquet is removed. We may always expect copious parenchymatous bleeding, partly owing to reaction after the elastic bandage, partly because the spray prevents, in a measure, the formation of clots. Arteries which have been overlooked during the previous ligation must now be secured; the rest of the bleeding can easily be stayed by compression or application of cold. The wound is then carefully washed out with carbolized water, no union of the edges is to be attempted until all hæmorrhage has ceased. The drainage-tubes are then to be introduced and the superficial sutures applied with antiseptic silk. The personal tact of the operator will show him where and how many drainage-tubes are necessary to facilitate the egress of the discharge. They should in all cases be first dipped in carbolized solution.

The wound is then covered with the protective, dipped in carbolized water, and over this by the lost gauze, that part of the gauze which is applied wet, as described above. Now the spray may be suspended, and the remainder of the gauze is applied.

Here I must digress to show the mode of dressing in wounds which were not made under the spray; after this the dressing is alike in all cases. In fresh wounds it is mostly sufficient to wash them out thoroughly with the carbolized solution. This must be done even in wounds of joints. Where suppuration has already commenced, there the carbolic acid is not sufficient to enforce asepsis. In these cases Lister uses the solution of chloride of zinc, with which the wound is carefully swabbed out. It should be forced into every recess by the pressure of the fingers. Still such cases are always doubtful, and if they do not result favorably the dressing is not always to blame, as the germs may have penetrated beyond the reach of the antiseptics.

We proceed now to the final dressing. A large antiseptic sponge is laid over the protective, and over it the *lost gauze* and the eight layers of gauze, the whole retained in place with broad gauze bandages, cut from the same material. They adapt themselves better than ordinary bandages, do not slip as easily, and have, also, antiseptic properties. A gentle, but firm, compression by them will facilitate the agglutination of the wound-surfaces and the egress of the discharge. The turns, especially where they are apt to be displaced, must be secured by small safety-pins. Inequalities of the surface, where the gauze does not closely adhere to the skin, must be filled with salicylic cotton, as such places are favorite passages for the wound-secretions.

The first dressing should be removed, at the latest, in twenty-four hours, sooner if the discharges appear anywhere on its surface. The changes are made under spray, which is carefully directed towards the wound, while pins, roller, gauze, sponge and protective are successively taken off. The protective will be found to have preserved its natural color, if the wound is aseptic. Where decomposition is going on, it shows dark, brownish spots, caused by the action of liberated sulphur in the pus upon the lead of the oiled silk. In this manner it is a delicate test of the success of the antisepsis. Whenever the protective is discolored, the wound must be treated like a septic wound, either with carbolized solution or the chloride of zinc. The spray is to cease as soon as the lost gauze covers again the protective. The changes have to be daily ones as long as the secretions are plentiful. The wound should be interfered with as little as possible,—the surroundings being gently cleaned with salicylic cotton or a sponge. If, as usually happens, the surface of the wound closes *per primam*, then two or three dressings will be sufficient. Besides the feelings of comfort or discomfort of the patient, and the staining of the dressing, we find the best indicator as to the necessity of changes in the temperature of the patient's body. Where this is normal, and the bandage unsoiled, we can pass on—everything is right. When the patient's temperature-slate shows an increase over the preceding observation, it will be an absolute indication to remove the dressing and examine the wound. In almost every instance we will find an accumulation of pus, which had not been reached by the drainage, or the tubes are choked, or some slight neglect had permitted some septic changes as shown by the spotted appearance of the protective. A longer drainage-tube, a counter-opening with a new tube, syringing with carbolic solution, will remedy the evil, and the temperature falls. The precautions must not be relaxed until the cicatrix is fully formed; a single unprotected granulation has given rise to violent erysipelas.

In conclusion of my remarks on the precautions necessary in the application of the dressings, I wish to state that the paraphernalia of the system are not as intricate and formidable as they appear on paper. In a few days they will become second nature, not only to the Surgeon, but to the nurses, even to the patient; the latter will not fail to call attention to the appearance of a stain, and the nurses will no more hand a dressing-forceps without first dipping it in the antiseptic.

This report would be incomplete without the description of a modification of Lister's dressings introduced by Professor Thiersch, in Leipzig. It consists, mainly, in the use of salicylic acid in place of the carbolic. Since the discovery made by Professor Kolbe, that salicylic acid can be cheaply prepared by the action of carbonic on carbolic acid, it has become available as an antiseptic. It is said to have the same properties as carbolic acid, without its irritating effects and unpleasant odor. The results attained by Thiersch seem as good as those of the carbolized dressing, and the method deserves a trial, especially as it combines with other advantages that of cheapness. In place of the gauze, Thiersch uses salicylic cotton; for the spray, salicylic solution (1 in 300); the same for washing of wounds and immersion of the sponges. The instruments are, however, oxidized by it, and should be kept in carbolic solution. The protective is not necessary, as the dressing is not irritating, and may be replaced by a piece of carbolized gauze.—A. C. Girard, *in his report to the Surgeon General U. S. A.*

